# IFN Regulatory Factor 1 Mediates Macrophage Pyroptosis Induced by Oxidized Low-Density Lipoprotein in Patients with Acute Coronary Syndrome

**DOI:** 10.1155/2019/2917128

**Published:** 2019-12-01

**Authors:** Min Guo, Rui Yan, Hongmei Yao, Liqin Duan, Meng Sun, Zheng Xue, Yongping Jia

**Affiliations:** ^1^Department of Cardiology, First Hospital of Shanxi Medical University, Taiyuan, China; ^2^Shanxi Medical University, Taiyuan, China

## Abstract

**Background:**

Atherosclerosis (AS) is recognized as a chronic inflammatory disease. It is caused by the interaction between inflammatory cells such as macrophages, dendritic cells, and lipoproteins. Evidence has revealed that macrophage pyroptosis in lesion contributes to the formation of the necrotic core and thinning of the fibrous cap, which plays crucial roles in the onset of acute coronary syndrome (ACS). IFN regulatory factor 1 (IRF-1) is a pleiotropic transcription factor involved in various immune processes and cell death. We propose that IRF-1 may be implicated in macrophage pyroptosis in the pathogenesis of AS and ACS.

**Methods:**

Patients with stable angina, unstable angina, acute myocardial infarction, and clinical presentation of chest pain were enrolled. The expression of IRF-1 in human PBMC-derived macrophages was analyzed. Then, overexpression and inhibition of IRF-1 was performed in macrophages from patients with ACS to explore the possible role and mechanism of IRF-1 involvement in macrophage pyroptosis.

**Results:**

The expression of IRF-1 in macrophages was upregulated in ACS patients. The overexpression or inhibition of IRF-1 effectively modulated caspase-1 activation, as well as macrophage lysis, expression of gasdermin D-N (GSDMD-N), production of IL-1*β* and IL-18, and activation of NLRP3-ASC inflammasome, which were all inhibited by caspase-1 inhibitor. Further experiments revealed that pyroptosis and the downstream inflammatory response in AS induced by IRF-1 is a process that is dependent on reactive oxygen species (ROS) generation.

**Conclusion:**

Our observations suggest that IRF-1 potently activates ox-LDL-induced macrophage pyroptosis and may play an important role in AS and ACS.

## 1. Introduction

Atherosclerosis (AS) is a complex chronic inflammation disease characterized by an excessive accumulation of lipids within the arterial wall and the activation of various immune cells, such as macrophages, monocytes, T lymphocytes, and dendritic cells [1–4]. Its clinical complications, such as coronary artery disease (CAD), stroke, and peripheral vascular disease, are the leading causes of morbidity and mortality worldwide [5, 6]. The deaths of endothelial cells, macrophages, smooth muscle cells, and many other types of vascular cells have been observed in human atherosclerotic plaques and play an important role in the instability of atherosclerotic lesions [7]. The death of macrophages in early atherosclerotic lesions attenuates plaque inflammation by decreasing the number of these cells and reducing the synthesis of matrix metalloproteinases (MMPs). However, the deaths of macrophages in late lesions may induce necrotic core formation and lead to the instability of atherosclerotic lesions, which resulting in the onset and development of acute coronary syndrome (ACS) [8, 9].

Pyroptosis is a unique form cell death that is dependent on caspase-1 and features cell membrane pores mediated by gasdermin D-N (GSDMD-N), DNA fragmentation, and the production of proinflammatory cytokines [10, 11]. Numerous studies have shown that a large majority of dying cells in human atherosclerotic lesions exhibit typical ultrastructure cell lysis, but not typical cell deaths such as apoptosis and necrosis [12–14]. A recent study reported that caspase-1 was activated in human advanced atherosclerotic plaques and colocalized with macrophages. Casepase-1 activation in macrophages results in processing the proinflammatory cytokines, IL-1*β* and IL-18, to their active forms and the death of the macrophages. In vitro experiments showed that ox-LDL induces caspase-1 activation and this activation is required for ox-LDL-induced macrophage lysis, as well as IL-1*β* and IL-18 production [15]. Until now, there has been increasing evidence supporting that pyroptosis may be involved in the instability of atherosclerotic lesions [16]. However, the underlying mechanism modulating caspase-1 activation during ox-LDL-induced macrophage pyroptosis remains obscure.

Transcription factors of the interferon regulatory factor family are recognized as interferon-induced transcription factors. These factors can efficiently participate in many proinflammatory and proinjury responses. Of the IRF members, at least 3 (IRF-1, IRF-5, and IRF-8) are involved in macrophage differentiation and polarization [17–19]. Recently, IRF-2 was found to be essential for the transcriptional activation of GSDMD and inducing pyroptotic cell death [20]. Extensive analysis of IRF-1 revealed that it plays essential roles in the development and function of specific cells of the immune system. Published reports demonstrated that IRF-1 is important for the antitumor efficacy of cyclophosphamide (CTX) and for the regulation of many immunomodulatory activities of CTX, such as Th1 polarization, Treg depletion, and inflammation [21]. Our previous studies have shown that IRF-1 participates in the onset and progression of ACS by modulating the function of Th1 cells and dendritic cells [22, 23]. Evidence has also emerged that IRF-1 may play an important role in cell death involving caspase-1 activation, which has been associated with oligodendrocyte pyroptosis in encephalomyelitis and multiple sclerosis [24–26]. Therefore, we hypothesized that IRF-1 may be implicated in the pathogenesis of AS by regulating macrophage pyroptosis and eventually leading to ACS.

In the current study, we showed that the expression of IRF-1 in human macrophages was upregulated in patients with ACS. The overexpression or silencing of IRF-1 expression in macrophages effectively promoted or attenuated the activation of caspase-1, as well as macrophage lysis, expression of GSDMD-N, and production of IL-1*β* and IL-18. In addition, we also revealed that pyroptosis and the downstream inflammatory response in AS induced by IRF-1 is a process that is activated by reactive oxygen species (ROS). These findings suggest that IRF-1 could potently activate ox-LDL-induced macrophage pyroptosis, which may play an important role in the progression and deterioration of ACS.

## 2. Materials and Methods

### 2.1. Patients and Control Subjects

The study protocol conformed to the ethical guidelines of the 1975 Declaration of Helsinki and was approved by the ethics committee of the First Hospital of Shanxi Medical University. Informed consent was obtained from all patients. Ninety-four patients with coronary disease were enrolled from the hospital from September 2017 to April 2018. The exclusion criteria were patients with a history of malignant tumor, advanced liver disease, renal failure, thromboembolism, valvular heart disease, dilated cardiomyopathy, and other inflammatory diseases. The patients were divided into four groups.

1. *CPS Group (16 Men and 6 Women)*. Clinical presentation of chest pain without any electrocardiographic changes, coronary artery stenosis, or coronary spasm when acetylcholine was given through intracoronary injection during arteriography
2. *SA Group (14 Men and 5 Women)*. Clinical presentation of discomfort in the chest, jaw, shoulder, back, or arm with downsloping or horizontal ST-segment depression >1 mm in an exercise test that can be relieved with nitroglycerin or rest
3. *UA Group (20 Men and 8 Women)*. Angina pectoris with an accelerating pattern, prolonged duration (>20 min), or recurrent episodes at rest or within minimal effort, with ischemic electrocardiographic changes such as ST-segment changes and/or T-wave inversion
4. *AMI Group (20 Men and 5 Women)*. Chest pain lasting >30 min within the 24 h before enrollment, that was not relieved by nitroglycerin, and muscular injury evidence by significantly increasing creatine kinase-MB and troponin I levels

### 2.2. Blood Samples and Cell Culture

Blood samples were drawn from all patients using 21-gauge needles for clean venipuncture of an antecubital vein on the morning after arrival. The time between the onset of chest pain and the performance of phlebotomy was <24 h. Blood was drawn into sodium heparin vacutainers (Becton Dickinson, Cedex, France) and then centrifuged for 15 min at 1000 × g. Peripheral blood monocyte cells (PBMCs) were isolated from peripheral blood samples by density gradient centrifugation (2000 rounds/min for 20 min) with Ficoll-Hypaque (Sigma Chemical Co., St. Louis, MO) to remove red blood cells and granulocytes as described in our previous reports [22, 23, 27]. CD14+ cells were isolated by using a CD14-positive cell isolation kit (Miltenyi Biotec, CA, USA). The purified cells were suspended at a density of 5 × 10^5^ cells/ml in RPMI-1640 (Sigma) supplemented with 10% fetal bovine serum (FBS, Sigma), 10 mmol/l glutamine, 50 U/ml penicillin, 50 *μ*g/ml streptomycin, and 20 ng/ml human GM-CSF (BD Biosciences, CA, USA) for 6 days at 37°C with 5% CO_2_ for 6 days to induce differentiation into macrophages. Cell purity was confirmed by flow cytometry (90-95%).

### 2.3. Lentiviral Vector Construction

The lentiviral vector PGC-FU-IRF-1 (GenBank Accession no. NM_012591.1) and IRF-1 shRNA were designed and synthesized as previously reported [23]. The IRF-1 shRNA sequences were obtained from Invitrogen (Invitrogen, Carlsbad, CA). The sequences were designed as previously described [22, 23]: IRF-1 shRNA sense: 5′-AAGTAATTTCCCTTCCTCATCTATAGTGAGTCGTATTAGGATCC-3′, and IRF-1 shRNA antisense: 5′-AAGATGAGGAAGGGAAATTACTATAGTGAGTCGTATTAGGATCC-3′. All lentiviral vectors were purified to a titer of 1 × 10^9^ TU/ml.

### 2.4. Synthesis of siRNA and Transfection

SiRNA was synthesized by Invitrogen (Invitrogen, Carlsbad, CA). The sequences of the siRNAs are listed in Table 1.

### 2.5. Cell Isolation and Gene Transfection In Vitro

One day before transfection, PBMC-derived macrophages from the ACS group were cultured in OMEM without serum and antibiotics. The cells (5 × 10^5^) were aliquoted into twelve-well culture plates and then divided into the following groups: ACS group, IRF-1 group (transfected with pGC-FU-IRF-1), IRF-1 shRNA group (transfected with pGC-FU-IRF-1 shRNA), and control shRNA group (transfected with pGC-FU-control shRNA). Twelve hours after transfection, we decanted the supernatants and added new RPMI-1640 medium with antibiotics. The transfected macrophages were cultured for 7 days, and then, expression due to stable transfection of IRF-1 and IRF-shRNA was obtained. After day 7, differentiated macrophages were cultured with Dil-ox-LDL (50 *μ*g/ml). Transfection of siRNA was performed at a concentration of 50 nM using Lipofectamine 2000.

### 2.6. Inhibition Experiments

For inhibition experiments, the cells were pretreated with caspase inhibitors (Ac-YVAD-CHO for caspase-1, Ac-DEVD-CHO for caspase-3, Z-IETD-CHO for caspase-8, Ac-LEHD-CHO for caspase-9, and z-VAD-CHO for pan-caspase; Life science, Germany) at 100 *μ*M concentrations for 1 h at 37°C prior to the initiation of the experiment.

### 2.7. Cytokine Detection

The cytokines (IL-1*β*, IL-18, IL-6, IL-33, and TNF-*α*) in the supernatants of the ACS macrophages and transfected macrophages cultured in RPMI-1640 medium containing Dil-ox-LDL (50 *μ*g/ml) were detected by enzyme-linked immunosorbent assay (ELISA) kits (Bender Medsystems, Burlingame, CA) according to the manufacturer's recommendations. Each sample was tested in triplicate.

### 2.8. Real-Time Quantitative Reverse Transcription PCR

Total cellular RNA from the human monocyte-derived macrophages or transfected macrophages was isolated using a TRIzol RNA extraction kit (Invitrogen, Carlsbad, CA, USA) according to the manufacturer's instructions. The RNA concentration and purity were measured by a spectrophotometer. 5 *μ*g of total RNA was reverse transcribed into cDNA using first-strand real-time polymerase chain reactions (Takara, Kyoto, Japan), and the resulting cDNA was used as a PCR template. GAPDH was used as an endogenous control. The PCR reaction mixture contained PCR buffer, SYBR Green Taq DNA polymerase, and the specific primers for GAPDH, IRF-1, caspase-1, IL-1*β*, IL-18, GSDMD-N, NLRP3, and ASC (Table 2) following the manufacturer's protocol. The mRNA levels were quantified by the relative quantification analysis using an ABI7500 system and SDS software (Applied Biosystems, Foster City, CA), and the copies of each mRNA were determined by the standard curved method.

### 2.9. Western Blot Analysis

Total proteins were extracted from human monocyte-derived macrophages or transfected macrophages after incubation with Dil-ox-LDL. The protein concentration was assessed by BCA method. 50 *μ*g protein per sample was heated to 99°C for 10 min and then separated on 6% to 12% SDS-polyacrylamide (SDS-PAGE) gels and then electrophoretically transferred to nitrocellulose (NC) membranes. The membranes were blocked with blocking buffer (5% nonfat milk in TBS containing 0.3% Tween-20) for 2 h, washed, and then incubated overnight at 4°C in a 1 : 2000 dilution of specific antibodies against IRF-1, caspase-1 p10, IL-1*β* p17, IL-18, GADPH, ASC (Abcam, Cambridge, UK), NLRP3, and GSDMD-N (Proteintech, Chicago, USA). After three washes, secondary antibodies were added for 2 h at room temperature. Finally, the membranes were washed three times, and the signal was detected by using ECL according to the manufacturer's protocols (Pierce, Rockford, IL). Protein levels were quantified by scanning the band intensities using NIH Image version 1.61.

### 2.10. Cell Death Assay

Pyroptotic cell death was evaluated by lactate dehydrogenase (LDH) release and EthD-III/calcein AM staining. LDH release in the supernatant was collected, and cytotoxicity was measured using the CytoTox 96 kit (Promega, USA). Total LDH release was obtained by lysis of macrophages with a solution of 0.1% Triton X-100, and the percentage cytotoxicity was calculated as 100 × ((experimental LDH release-spontaneous LDH release)/(maximum LDH release-spontaneous LDH release)).

For EthD-III/calcein AM staining, macrophages (10^5^ cells/well) were cultured in a 12-well plate and pretreated with lentiviral vectors and Dil-ox-LDL. The virtually nonfluorescent cell permeable calcein AM is converted to the intensely fluorescent calcein, which produced green fluorescence. EthD-III enters dead cells and produces red fluorescence. Pyroptotic dead cells were assessed by counting the ratio of red-positive cells to the total number of cells.

### 2.11. Analysis of DNA Fragmentation

Nuclear DNA fragmentation was detected by TUNEL staining using a cell death detection kit (Promega, USA) as directed by the manufacturer's instructions. Samples were counterstained with DAPI and analyzed with a fluorescence microscope. The TUNEL-positive nuclei (green) index was determined by dividing the number of cells with DNA-fragmented nuclei by the total nuclei population. Analysis was carried out randomly in 5 fields of each section.

### 2.12. Caspase-1 Activity Assay

Caspase-1 activity was measured by using colorimetric assay (Biovision, Mountain View, CA). Cells were lysed in lysis buffer containing 50 mM HEPES (pH 7.4), 0.1% 3-[(3-cholamidopropyl)-dimethylammonio]-1-propanesulfonate, and 5 mM dithiothreitol. 50 *μ*g of total cytosolic protein was used to assess cytosolic caspase-1 activity. This mixture was placed in a 96-well plate and incubated for up to 4 h at 37°C with caspase-1 substrate conjugated to the chromophore p-nitroanilide (pNA). The pNA light emission was quantified by spectrophotometry at 405 nm.

### 2.13. Detection of Intracellular ROS Levels

The level of intracellular ROS was detected by reactive oxygen species assay kit (Beyotime, China) according to the manufacturer's instructions. Macrophages were incubated with PBS or Dil-ox-LDL in the presence or absence of N-acetyl-cysteine-cysteine (NAC) for 24 h and were loaded with DCFH-DA (10 *μ*M) at room temperature for 30 min in the dark. The fluorescence intensity of five fields was examined, and the ROS level was measured by fluorescence intensity.

### 2.14. Statistical Analysis

All statistical analyses were performed with SPSS15.0. The results are expressed as the mean ± SD of at least three independent experiments. The significance of difference was assessed by ANOVA and the Newman-Keuls test. *p* < 0.05 was considered statistically significant.

## 3. Results

### 3.1. Basic Clinical Characteristics of Patients

The basic clinical characteristics of the patients are shown in Table 3. There were no significant differences in age, risk factors, and medications among the patients with CPS, SA, UA, and AMI.

### 3.2. Relative Expression of IRF-1 in PBMC-Derived Macrophages

**Figure 1:**
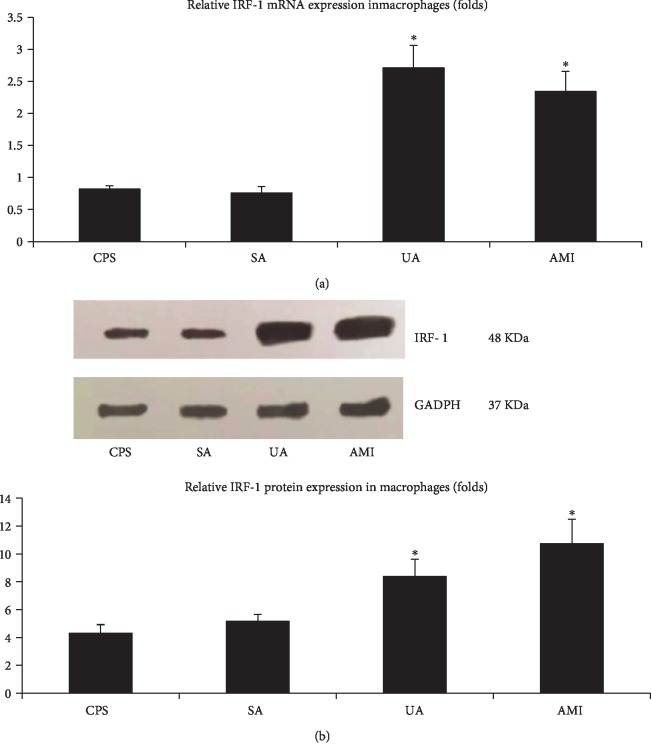
The relative mRNA and protein expression of IRF-1 in human PBMC-derived macrophages. (a) The relative IRF-1 mRNA expression was determined by real-time PCR. (b) The relative IRF-1 protein was determined by western blot. ∗ indicates vs CPS and SA group (^∗^*p* < 0.05). # indicates vs CPS group (^#^*p* > 0.05).

The relative mRNA and protein expression of IRF-1 were investigated in PBMC-derived macrophages from patients with CPS, SA, UA, and AMI. The relative IRF-1 mRNA and protein expression in the ACS groups (including AMI and UA) were significantly elevated compared with those of the SA and CPS groups. Thus, there was no significant difference between the SA and CPS groups (Figure 1).

### 3.3. IRF-1 Promotes Caspase-1 Activity in Macrophages

**Figure 2:**
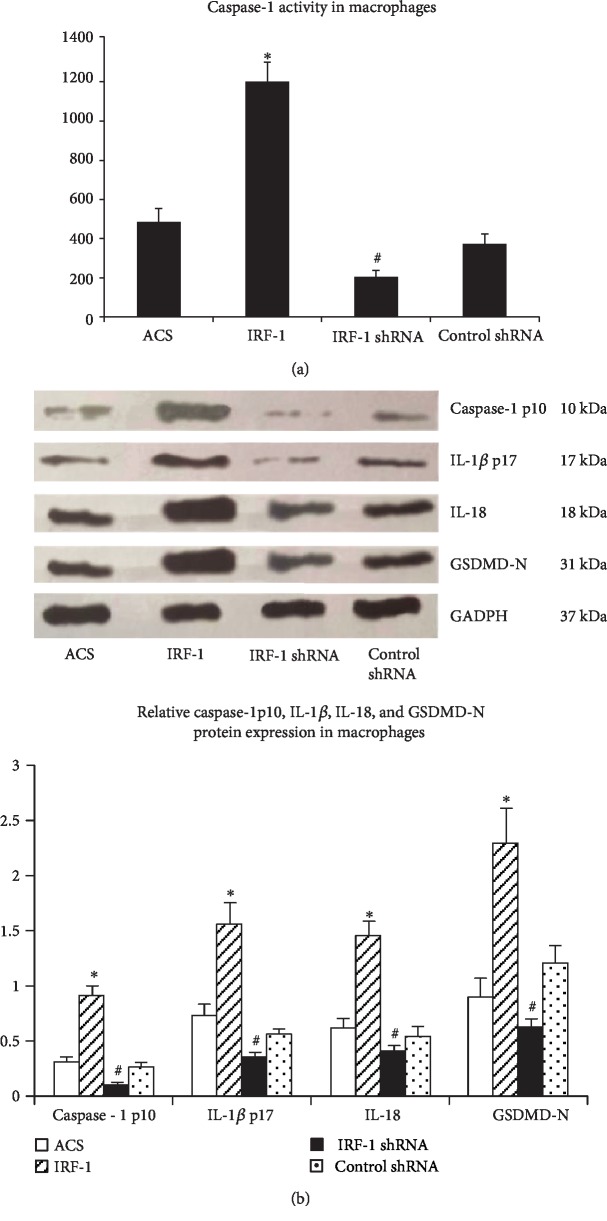
IRF-1 promotes caspase-1 activity in macrophages. Human macrophages were divided into four groups (group 1: cultured with PBS as a control group; group 2: transfected with pGC-FU-IRF-1, group 3: transfected with IRF-1 shRNA; and group 4: transfected with control shRNA) and then cultured with Dil-ox-LDL (50 *μ*g/ml) for 48 h. (a) Caspase-1 activity was measured using the colorimetric assay. ∗ indicates vs ACS and control shRNA group (^∗^*p* < 0.01). # indicates vs ACS group and control shRNA (^#^*p* < 0.05). (b) Western blot analysis demonstrated that the protein levels of caspase-1 p10, GSDMD-N, IL-1*β* p17, and IL-18 increased in the IRF-1 group (*p* < 0.05). ∗ indicates vs ACS and control shRNA group (^∗^*p* < 0.01). # indicates vs ACS group and control shRNA (^#^*p* < 0.05).

To unravel the exact mechanism of caspase-1 activation in macrophages, we aliquoted macrophages from ACS patients into wells and classified them into four groups: ACS group, IRF-1 group (transfected with pGC-FU-IRF-1), IRF-1 shRNA group (transfected with pGC-FU-IRF-1 shRNA), and control shRNA group (transfected with pGC-FU-control shRNA). Then, the transfected cells were treated with Dil-ox-LDL (50 *μ*g/ml) for 48 h. Caspase-1 activation was measured by using caspase-1 substrate conjugated to the chromophore pNA (Ac-YVAD-pNA) and western blot. As shown in Figure 2(a), caspase-1 activity was significantly upregulated in the IRF-1 group compared with the ACS and control shRNA groups. Western blot analysis demonstrated that the protein levels of caspase-1 p10, GSDMD-N, IL-1*β* p17, and IL-18 increased in the IRF-1 group. In contrast, caspase-1 activity and the protein levels of caspase-1 p10, GSDMD-N, IL-1*β* p17, and IL-18 were obviously decreased in the IRF-1 shRNA group (Figure 2(b)).

### 3.4. IRF-1 Induces Pyroptotic Macrophage Death Which Is a Process Dependent on Caspase-1 Activation

**Figure 3:**
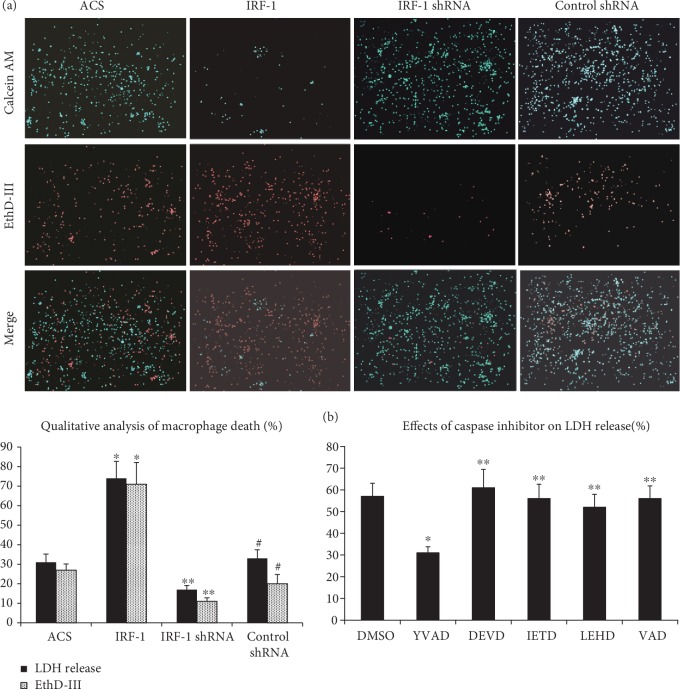
IRF-1-induced macrophage pyroptosis is caspase-1 dependent. Cell death was visualized by EthD-III (red)/calcein AM (green) staining (100x) or LDH release. (a) Qualitative analysis of cell lysis expressed as the percentage of EthD-III-positive cells or LDH release in the total cells. ∗ indicates vs ACS and control shRNA group (^∗^*p* < 0.01). ∗∗ indicates vs ACS and control shRNA group (^∗^*p* < 0.05). # indicates vs ACS group (^#^*p* < 0.05). (b) Cells were treated with DMSO or caspase inhibitors (Ac-YVAD-CHO for caspase-1, Ac-DEVD-CHO for caspase-3, Z-IETD-CHO for caspase-8, Ac-LEHD-CHO for caspase-9, and z-VAD-CHO for pan-caspase). ∗ indicates vs DMSO group (^∗^*p* < 0.01). ∗∗ indicates vs DMSO (∗∗*p* < 0.05).

There is evidence indicating that pyroptosis is a form of cell lysis which is dependent on caspase-1. We measured whether IRF-1 could induce pyroptotic macrophage death by LDH release and EthD-III/calcein AM staining. The results showed that the LDH release and extensive EthD-III-positive cells were significantly increased in the IRF-1 group compared with the ACS and control-shRNA groups. Conversely, IRF-1 shRNA did obviously reduce LDH release and the number of EthD-III-positive cells (Figure 3(a)).

Next, to further explore whether caspase-1 is involved in IRF-1-induced macrophage lysis, we performed an inhibitory experiment. Macrophages were pretreated with DMSO or caspase inhibitors for 1 h and then incubated with ox-LDL for 48 h. Our study showed the caspase-1 inhibitor strongly suppressed cell lysis after transfection with pGC-FU-IRF-1 compared with that of the DMSO group, whereas the difference between other apoptotic caspase (caspase-3, caspase-8, and caspase-9) inhibitors and the DMSO group was not significant (Figure 3(b)). Based on these data, we considered that IRF-1 could dramatically induce macrophage pyroptosis and that caspase-1 activation was involved in IRF-1-induced macrophage lysis.

### 3.5. IRF-1 Modulates the Secretion of Inflammation Cytokines by Macrophages

**Figure 4:**
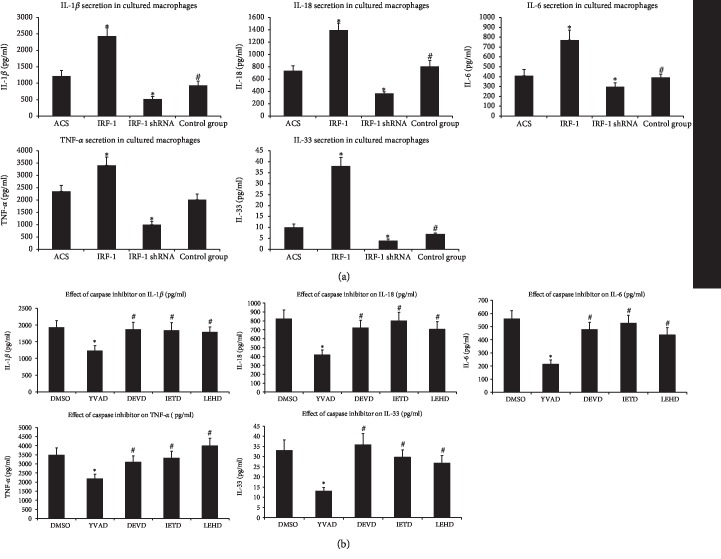
IRF-1 modulates the secretion of inflammation cytokines by macrophages. The secretion of cytokines was assayed by ELISA. (a) Macrophages were transfected with pGC-FU-IRF-1, IRF-1 shRNA, and control-shRNA; ACS group was cultured with PBS. Then, the cells were cultured with ox-LDL for 48 h. The IL-1*β*, IL-18, IL-6, IL-33, and TNF-*α* concentrations in the supernatants of the transfected macrophages were detected. ∗ indicates vs ACS and control shRNA group (^∗^*p* < 0.01). # indicates vs ACS group (^#^*p* > 0.05). (b) After transfected with pGC-FU-IRF-1, the cells were treated with DMSO or caspase inhibitors for 1 h and then stimulated with ox-LDL for 48 h. ∗ indicates vs DMSO group (^∗^*p* < 0.01). # indicates vs DMSO group (^#^*p* > 0.05).

Our results showed that the cytokine concentrations (IL-1*β*, IL-18, IL-6, IL-33, and TNF-*α*) in the culture supernatant of transfected cells stimulated with ox-LDL were significantly increased in the IRF-1 group compared with the ACS group. Additionally, the secretion of these cytokines was obviously decreased in the IRF-1 shRNA group (Figure 4(a)).

To determine whether caspase-1 participates in the upregulation of these cytokines in macrophages, we performed an experiment with inhibitors. Our results showed that the caspase-1 inhibitor exhibited a strong suppression of inflammatory cytokine secretion after transfection with pGC-FU-IRF-1 compared with that of the DMSO group, whereas the difference between the other apoptotic caspase (caspase-3, caspase-8, and caspase-9) inhibitors and the DMSO group was not significant (Figure 4(b)).

### 3.6. IRF-1 Is Involved in ox-LDL-Induced DNA Fragmentation in Macrophages

**Figure 5:**
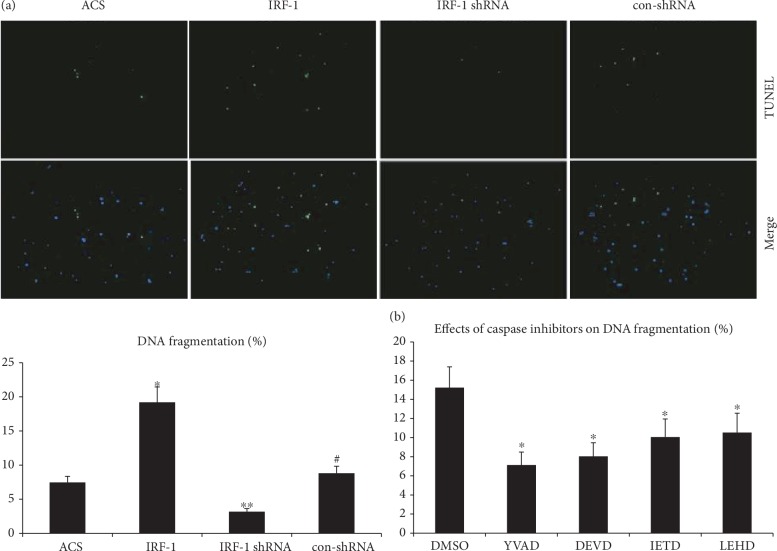
IRF-1 is involved in ox-LDL-induced DNA fragmentation in macrophages. DNA fragmentation was measured by TUNEL staining (green) (100x). (a) Cells were aliquoted into four groups as described above. Qualitative analysis of DNA fragmentation was performed by randomly counting 5 fields and is expressed as a percentage of the total nuclei cells. ∗ indicates vs ACS group (^∗^*p* < 0.01). ∗∗ indicates vs ACS and IRF-1 group (^∗∗^*p* < 0.01). # indicates vs ACS group (^#^*p* > 0.05). (b) After transfected with pGC-FU-IRF-1, the cells were incubated with caspase inhibitors for 3 h. ∗ indicates vs DMSO group (^∗^*p* < 0.01).

Recent studies have demonstrated that pyroptosis is a process characterized by cell lysis, inflammation, and DNA fragmentation. In order to further characterize IRF-1-induced DNA fragmentation in macrophages, we assessed the effect of IRF-1 on DNA fragmentation using TUNEL reaction. We found that a TUNEL-positive reaction was observed in ox-LDL-induced cell death. IRF-1 largely induced DNA fragmentation, whereas IRF-1 shRNA showed a suppressive effect (Figure 5(a)). After pretreatment with caspase inhibitors, we found that caspase-1, caspase-3, caspase-6, and caspase-9 inhibitors all had suppressive effects on IRF-1-induced DNA fragmentation, suggesting that both pyroptotic and apoptotic pathways participate in this process (Figure 5(b)).

### 3.7. Effects of IRF-1 on the NLRP3-ASC Inflammasome in Macrophages

**Figure 6:**
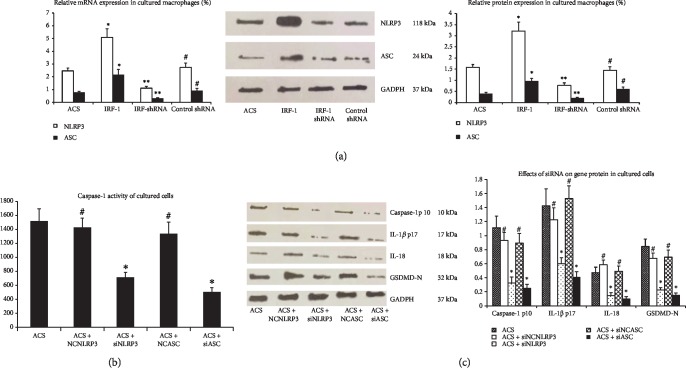
Effects of IRF-1 on the NLRP3-ASC inflammasome in macrophages. (a) Macrophages from ACS patients were aliquoted into four groups and treated with Dil-ox-LDL. Relative mRNA and protein levels of NLRP3 and ASC were measured by RT-PCR and western blot. ∗ indicates vs ACS group (^∗^*p* < 0.01). ∗∗ indicates vs ACS and IRF-1 group (^∗∗^*p* < 0.01). # indicates vs ACS group (^#^*p* > 0.05). (b) Macrophages were transfected with pGC-FU-IRF-1 for 36 h, and then, cells were transfected with nontargeting siRNA and siRNA specific for NLRP3 and ASC. Caspase-1 activity was detected by Ac-YVAD-pNA. ∗ indicates vs ACS group (^∗^*p* < 0.01). # indicates vs ACS group (^#^*p* > 0.05). (c) Macrophages were transfected with NLRP3 siRNA specific or ASC siRNA after transfection with pGC-FU-IRF-1 as described. Protein of caspase-1 p10, IL-1*β* p17, IL-18, and GSDMD-N were measured by western blot. ∗ indicates vs ACS group (^∗^*p* < 0.01). # indicates vs ACS group (^#^*p* > 0.05).

Inflammasomes are multiprotein complexes that are involved in the activation of caspase-1. An inflammasome complex is generally composed of the NLRP family, procaspase-1, and the adaptor protein ASC [28, 29]. The crucial role of the inflammasome including ASC and NLRP3 in atherosclerosis is the most studied [30]. As depicted in Figure 6(a), the mRNA and protein expression of NLRP3 and ASC were upregulated in the IRF-1 group. Conversely, IRF-1 shRNA obviously reduced the expression of NLRP3, caspase-1, and ASC.

Moreover, silencing NLRP3 and ASC significantly suppressed the activation of caspase-1, the expression of GSDMD-N, and the production of IL-1*β* and IL-18 induced by IRF-1 (Figure 6(b)).

### 3.8. IRF-1-Induced Human Macrophage Pyroptosis Requires ROS

**Figure 7:**
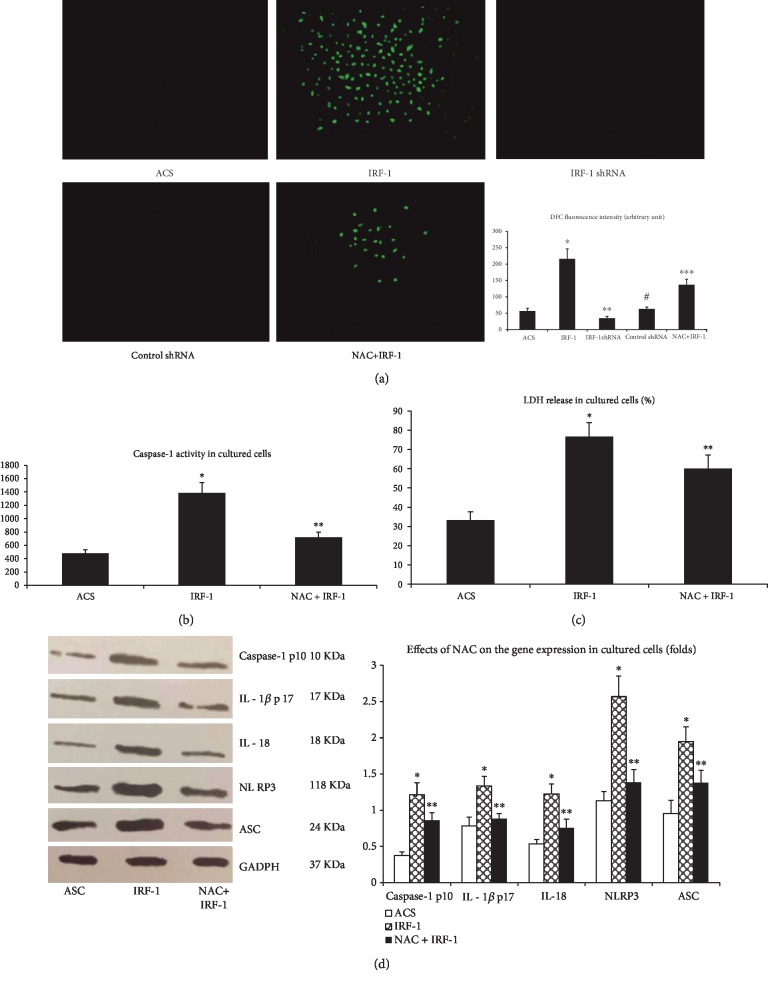
IRF-1-induced human macrophage pyroptosis requires ROS. (a) Macrophages from ACS patients were cultured with ox-LDL (48 h) and then divided into five groups in which three groups were transfected with pGC-FU-IRF-1, pGC-FU-IRF-shRNA, and pGC-FU-control-shRNA and one group was pretreated with NAC (10 nM, 24 h) and then were transfected with pGC-FU-IRF-1. Intracellular ROS were assessed by DCFH2-DA (200x). ∗ indicates vs ACS group (^∗^*p* < 0.01). ∗∗ indicates vs ACS and IRF-1 group (^∗∗^*p* < 0.01). # indicates vs ACS group (^#^*p* > 0.05). ∗∗∗ indicates vs IRF-1 group (^∗∗∗^*p* < 0.05). (b) Caspase-1 activity was measured by Ac-YVAD-pNA. (c) Cell death was measured by the percentage of LDH release. (d) Protein expression of NLRP3, ASC, caspase-1 p10, IL-1*β* p17, and IL-18 was measured by western blot. ∗ indicates vs ACS group (^∗^*p* < 0.01). ∗∗ indicates vs ACS and IRF-1 group (^∗∗^*p* < 0.01).

Recent studies demonstrated that ROS are involved in inflammasome activation [31]. The role of ROS in IRF-1-induced pyroptosis in human macrophages was measured. The overexpression of IRF-1 stimulated intracellular ROS generation compared with that of the ACS group, and this effect was abrogated by ROS inhibitors, such as N-acetyl-cysteine-cysteine (NAC) (Figure 7(a)). NAC also inhibited IRF-1-induced caspase-1 activity, cell lysis, and the expression of NLRP3, ASC, caspase-1 p10, IL-1*β* p17, and IL-18 (Figure 7(b)). These results indicate that IRF-1-induced macrophage pyroptosis was dependent on ROS generation.

## 4. Discussion

Atherosclerosis which underlies the leading cause of death is a complex inflammatory chronic disease and may eventually lead to acute, occlusive luminal thrombosis and its consequences. Pyroptosis is a novel form of inflammatory cell death that plays a pivotal role in the pathogenesis of various cardiovascular diseases, including myocardial infarction (MI), hypertension, cardiomyopathy, heart failure (HF), and AS. To our knowledge, in atherosclerotic heart disease, an increased number of cell deaths exist at various stages of human atherosclerotic plaques. The death of macrophages, smooth muscle cells, and other types of cells has been confirmed to play an important role in advanced lesions [32]. However, the exact link between macrophage pyroptosis and AS/ACS has not been elucidated.

Our previous study reported that IRF-1 was involved in the progression of AS/ACS by modulating the function of Th1 cells and dendritic cells (DCs) [22, 23]. IRF-1 which is a pleiotropic transcription factor participates in pyroptotic cell death. Caspase-1 contains an IRF-1-binding element, and the caspase-1 activity can be directly modulated by IRF-1 in the development of multiple sclerosis and acute lung injury [33–35]. Therefore, we explored the novel role of IRF-1 in macrophage pyroptosis in ACS patients. Our results showed that the expression of IRF-1 in PBMC-derived macrophages was higher in patients with ACS compared with patients with SA and CPS. Furthermore, we found that ox-LDL promoted caspase-1 activation in macrophages and that IRF-1 could potentially induce caspase-1 activation, cell lysis, cytokine secretion, and inflammasome expression in macrophages after pretreatment with ox-LDL. These findings indicate that IRF-1 may play an important role in the pathogenesis of ACS.

Pyroptosis is a proinflammatory form of regulated cell death which seems to be a combination of apoptotic and necrotic cell death and is dependent on the enzymatic activity of caspases. As far as we know, caspases are an evolutionarily conserved family of cysteine proteases that are involved in cell death [36]. The role of apoptotic caspases (caspase-3, caspase-8, and caspase-9) has been well studied. Sequence analysis suggests that three human caspases (caspase-1, caspase-4, and caspase-5) and three murine caspases (caspase-1, caspase-11, and caspase-12) have been categorized as the inflammatory caspase subfamily. Activated caspase-1 mediates cleavage of the cytosolic protein GSDMD and promotes the formation of membrane pores with an inner diameter of 10-24 nm and the release of cytoplasmic content into the extracellular space. Pyroptotic cells undergo nuclear condensation and DNA fragmentation and show TUNEL staining [37, 38]. This caspase-1-regulated cell death mode frequently coincides with the secretion of IL-1*β* and IL-18 [39–41]. In addition to caspase-1, the inflammatory caspases in humans (caspase-4 and caspase-5) as well as caspase-11 in rodents, which was confirmed as the ortholog of human caspase-4 and caspase-5, are incapable of cleaving pro-IL-1*β* and pro-IL-18, but each has been shown to directly cleave GSDMD and induce pyroptosis [40, 41]. The interdomain cleavage of GSDMD is a reliable marker for the activation of inflammatory caspases and cell pyroptosis.

A considerable body of evidence suggests that ox-LDL, which is abundantly deposited in vessels, plays an important role in the pathogenesis of AS. Many studies have indicated that ox-LDL expresses many morphological features characteristic of apoptotic and nonapoptotic cell death. In our study, we also found that ox-LDL effectively induced the activation of caspase-1. Furthermore, we also investigated the function of IRF-1 in the activation of caspase-1 and cell lysis in macrophages by inducing or silencing the expression of IRF-1. We demonstrated that caspase-1 activity and cell lysis were significantly upregulated after transfection with pGC-FU-IRF-1. Here, we also found that overexpression of IRF-1 in macrophages significantly induced the protein expression of caspase-1 p10, GSDMD-N, IL-1*β* p17, and IL-18. In contrast, inhibition of IRF-1 decreased the caspase-1 activity, cell lysis, and the protein levels of caspase-1 p10, GSDMD-N, IL-1*β* p17, and IL-18. These results indicate that IRF-1 may modulate macrophage pyroptosis by activating caspase-1 and then mediate the cleavage of GSDMD and the secretion of IL-1*β* and IL-18 and that IRF-1 plays a pivotal role in plaque instability and vulnerability. In addition, some studies have demonstrated that caspases play important roles in initiating apoptosis and pyroptosis but are not involved in other programmed cell death processes [42]. Apoptosis in atherosclerosis is well documented as an active programmed process of autonomous cellular dismantling including activation of apoptotic caspase-3, caspase-8, and caspase-9 [43]. We identified that inhibitors of apoptotic caspase failed to block cell lysis and inflammatory cytokine secretion in human macrophages pretreated with ox-LDL and pGC-FU-IRF-1, suggesting that the nonapoptotic pathway may participate in IRF-1-induced macrophage death.

IRF-1 may also control the secretion of inflammatory mediators. It has been reported that caspase-1 activation triggers inflammatory cell death characterized by membrane rupture and the release of inflammatory factors other than IL-1*β* and IL-18. The present study showed that IRF-1 induced the secretion of inflammatory cytokines including IL-33, TNF-*α*, and IL-6. To determine whether caspase-1 participates in these upregulated cytokines in macrophages, we performed an inhibitory experiment with caspase inhibitors. Here, we validated that caspase-1 was required for IL-33, TNF-*α*, and IL-6 production. These results suggest that IRF-1 plays a critical role in pyroptosis-associated cytokines.

Caspases are activated in response to many stimuli including distinct inflammasomes. The canonical inflammasome is the cytosolic platform that activates caspase-1 in response to PAMPs or DAMPs [44]. Canonical inflammasomes are composed of accessory proteins and the nucleotide-binding oligomerization domain-like receptor (NLR) family. NLRP3 is the best studied inflammasome that can be activated by various agonists [45–47]. Afrasysb et al. found that the NLRP3 inflammasome is associated with the severity and prognosis of coronary atherosclerosis in patients with ACS. Our study showed that IRF-1 increased the expression of NLRP3 and suggests that the NLRP3 inflammasome pathway is involved in IRF-1-induced caspase-1 activation.

The generation of ROS is an essential mechanism implicated in NLRP3 inflammasome activation [31]. To explore whether a similar mechanism applies to NLRP3 inflammasome activation that occurs in IRF-1-induced human macrophage pyroptosis, we determined that overexpression of IRF-1 promoted intracellular ROS generation, and this effect was neutralized by ROS inhibitors such as NAC. Our results showed that pretreatment of cells with NAC could diminish IRF-1-induced caspase-1 activity, cell lysis, and the expression of NLRP3, ASC, caspase-1 p10, IL-1*β* p17, and IL-18, suggesting that IRF-1-induced macrophage pyroptosis was dependent on ROS generation.

In conclusion, our study revealed that the expression of IRF-1 may positively promote plaque rupture and the initiation of ACS via induction of macrophage pyroptosis. This newly discovered function of IRF-1 may provide a new therapeutic target for the development of AS and the onset of ACS.

## Figures and Tables

**Table 1:** Sequences of siRNA.

Gene	Sequence
NLRP3 sense	CAACAGGAGAGACCUUUAUTT
NLRP3 antisense	AUAAAGGUCUCUCCUGUUGTT
ASC sense	UCGCGAGGGUCACAAACGUTT
ASC antisense	ACGUUUGUGACCCUCGCGATT

**Table 2:** Primers of genes.

Gene	Sequence
IRF-1 sense	CCAGAGCAGGAACAAGGG
IRF-1 antisense	GGTCATCAGGCAGAGTGGA
Caspase-1 sense	CCTTAATATGCAAGACTCTCAAGGA
Caspase-1 antisense	TAAGCTGGGTTGTCCTGCACT
IL-1*β* sense	TACCTGTCCTGCGTGTTGAA
IL-1*β* antisense	TCTTTGGGTAATTTTTGGGATCT
IL-18 sense	TGCATCAACTTTGTGGCAAT
IL-18 antisense	ATAGAGGCCGATTTCCTTGG
GSDMD-N sense	GAGTGTGGCCTAGAGCTGG
GSDMD-N antisense	GGCTCAGTCCTGATAGCAGTG
NLRP3 sense	CACCTGTTGTGCAATCTGAAG
NLRP3 antisense	GCAAGATCCTGACAACATGC
ASC sense	AGGCCTGCACTTTATAGACC
ASC antisense	GCTGGTGTGAAACTGAAGAG
GADPH sense	GGATTGGTCGTATTGGG
GADPH antisense	GGAAGATGGTGATGGGATT

**Table 3:** Clinical characteristics of the enrolled participants.

Characteristics	CPS (*n* = 22)	SA (*n* = 19)	UA (*n* = 28)	AMI (*n* = 25)
Age (years)	58.6 ± 8.1	56.4 ± 7.5	61.9 ± 6.2	58.1 ± 8.4
Sex (male/female)	13/9	13/6	15/13	15/10
Risk factors				
Hypertension	8 (36%)	8 (42%)	14 (50%)	11 (44%)
Diabetes	5 (22.7%)	4 (21.1%)	8 (28.6%)	6 (24%)
Hyperlipidaemia	10 (45.5%)	10 (52.6%)	14 (50%)	15 (60%)
Tobacco	10 (45.5%)	11 (57.9%)	13 (46.4%)	13 (52%)
Medication (%)				
*β*-Blockers	12 (54.5%)	10 (52.6%)	18 (64%)	15 (60%)
Statins	17 (77.3%)	16 (84.2%)	24 (85.7%)	20 (80%)
Nitrates	15 (68.1%)	14 (73.7%)	22 (78.5%)	20 (80%)

## Data Availability

The data used to support the findings are available from the corresponding author.
